# Cul4a as a New Interaction Protein of PARP1 Inhibits Oxidative Stress-Induced H9c2 Cell Apoptosis

**DOI:** 10.1155/2019/4273261

**Published:** 2019-04-17

**Authors:** Ning Ye, Naijin Zhang, Ying Zhang, Hao Qian, Boquan Wu, Yingxian Sun

**Affiliations:** Department of Cardiovascular Medicine, The First Hospital of China Medical University, Shenyang 110001, China

## Abstract

Oxidative stress plays a major part in myocardial reperfusion injury. Cul4a is the core protein of CRLs E3 ubiquitin ligase complex; while it is known that Cul4a is responsible for various cancers, its role in cardiac function remains unclear. Hence, we have shown the protective function of Cul4a and its protection mechanism in oxidative stress-induced H9c2 cardiomyocyte apoptosis. Here, oxidative stress was induced by hydrogen peroxide (H_2_O_2_), CCK-8 assay and flow cytometry were used to analyze cell viability and apoptosis rate, western blot and immunofluorescence were used to quantitatively analyze the expression of protein, ROS fluorescence kit was used to detect reactive oxygen species (ROS) formation, and coimmunoprecipitation was used to identify protein interaction. In the results, it was found that Cul4a was involved in oxidative stress-induced H9c2 cell apoptosis and could inhibit H_2_O_2_-induced ROS generation and H9c2 cell apoptosis. Furthermore, we identified that when combining with PARP1, Cul4a could reduce its expression, and the interaction was enhanced under oxidative stress. In conclusion, our results indicate that Cul4a is a new protective factor involved in oxidative stress-induced cardiomyocyte injury and functions by tying and decreasing overactivated PARP1.

## 1. Introduction

Acute myocardial ischemia (AMI) is a main cause of death in China [1], and the most effective treatment is to perform myocardial reperfusion as soon as possible. However, this process can lead to myocardial cell dysfunction and apoptosis, which is called myocardial reperfusion injury [2]. Oxidative stress participates in the pathological process of nearly all cardiovascular diseases [3] and is crucial in myocardial reperfusion injury [4]. Therefore, exploring the mechanism of oxidative stress to alleviate myocardial cell damage is the key to the treatment of myocardial reperfusion injury.

PARP (poly ADP-ribose polymerase) is a DNA repair enzyme which is a cleavage substrate for caspase. It was considered to play an important role in DNA damage repair and apoptosis in the past. However, during oxidative stress, excessive activation of PARP1 can lead to depletion of ATP, which causes apoptosis [5]. Recently, several studies have also shown that PARP1 participates in oxidative stress-related cardiovascular diseases, including ischemia reperfusion injury [6, 7]. And the inhibitors of PARP1 can effectively relieve reperfusion injury in vivo experiment [8].

Cullin 4a (Cul4a) is a 87 kDa protein with a gene located at 13q34 in the high expression region of the oncogene. It is a scaffold protein of the modular, multisubunit E3 ubiquitin ligase complex, which dynamically combines and degrades protein in a periodic manner [9]. Abnormal expression of Cul4a is closely related to various cancers [10]. Cul4a is involved in a series of processes such as cell cycle, DNA damage repair, histone methylation, signaling pathways, and oncoprotein transfer [11]. However, both the function of Cul4a in the cardiovascular disease and its role in myocardial injury caused by oxidative stress are still unclear.

Here, we describe a novel role for Cul4a as a protector of myocardial cell apoptosis elicited by oxidative stress. Besides, Cul4a can reduce ROS generation, which is related to oxidative stress. We find that PARP1 is a new binding protein for Cul4a, and the mechanism that Cul4a can alleviate oxidative stress-induced cardiomyocyte injury may be associated with the degradation of overactivated PARP1.

## 2. Materials and Methods

### 2.1. Cell Culture and Treatment

H9c2, a clonal heart muscle cell line, which was from the Cell Bank of the Chinese Academy of Sciences (Shanghai, China), was cultured in high glucose DMEM with 10% FBS (GE Healthcare HyClone, USA) and 1% penicillin/streptomycin at 37°C in a 5% CO_2_ incubator. Oxidative stress was induced by hydrogen peroxide (H_2_O_2_); it is a recognized method.

### 2.2. Antibodies and Reagents

Antibodies to polyclone rabbit anticaspase3, monoclonal rabbit anti-PARP1, and mouse anti-Flag were obtained from Cell Signaling Technology (USA); polyclonal mouse anti-Cul4a, monoclonal rabbit anti-*α*-tubulin, and anti-GAPDH were obtained from Proteintech (Wuhan, China). Protein A/G magnetic beads were acquired from Biotool (Shanghai, China).

### 2.3. RNA Interference and Gene Overexpression

For Cul4a knockdown, 70% confluence H9c2 cardiomyocytes were transfected with control siRNA and Cul4a siRNA was provided by GenePharma (Shanghai, China). jetPRIME transfection reagent from PolyPlus (France) was used as the media to following the instructions. In order to preclude off-target effects, we synthesized three sequences and Western blot was used to ensure the efficiency of Cul4a knockdown. The target sequences were as follows: Cul4a siRNA-1: GCCUAGAGCUGUUUAGGAATT, Cul4a siRNA-2: GCGAGUACAUCAAGACCUUTT, and Cul4a siRNA-3: GCUGCUAUAGUCAGAAUAATT.

For Cul4a overexpression, the H9c2 cardiomyocytes were transfected with pCDNA3.1-3×Flag or pCDNA3.1-Cul4a-Flag provided by Sangon Biotech (China, Shanghai). Firstly, the medium was replaced with OPTI-MEM from Gibco (USA), after that, Lipofectamine 3000 from Invitrogen (USA) was used for transfection following the instructions.

### 2.4. Cell Viability Assay

Cell viability was calculated by Cell Counting Kit-8 (CCK-8) assay from Dojindo (Japan). The steps were as follows: cells were transfected in different groups for 48 hours, treated with normal media or H_2_O_2_ (50, 100, 200, and 400 *μ*M) in 2 hours, finally mixed with 100 *μ*l CCK-8 solution (1 : 10), and incubated for 1 h in 37°C after washing by PBS. Absorbance was measured at 450 nm by a microplate reader from Bio-Rad Laboratories (USA). Statistical software was used to calculate the cell survival viability.

### 2.5. Flow Cytometry Annexin-FITC/PI

Annexin V-fluorescein isothiocyanate (FITC) and propidium iodide (PI) assay were used to evaluate cell injury according to the instructions of the manufacturer. Cells were seeded in 6-well plates, and different groups were transfected for 48 hours following the experimental requirements. After transfection, cells were treated with normal media or H_2_O_2_ (200 *μ*M) for 2 hours. And then collecting cells, which were used by 0.25% trypsin without EDTA, were washed by PBS twice. Next, the cells were incubated in 500 *μ*l binding buffer with 5 *μ*l Annexin V and 5 *μ*l PI solution for 30 minutes at room temperature in the dark. Finally, the cells were analyzed by the FACSCalibur flow cytometry with FL-1 and FL-2 channel.

### 2.6. Immunofluorescence Analysis

Cellular immunofluorescence was used following the well-established procedure. In short, the cells of different groups were washed with PBS, fixed with 4% paraformaldehyde, and handled with 1% Triton X-100 for 20 minutes. After blocking for 30 minutes, the cells were incubated with the primary antibody of Cul4a (1 : 200) for 4 hours in room temperature and then the fluorescent secondary antibody (1 : 200) provided by Life Technologies (USA) was added. The nucleus was stained with DAPI for 3 minutes avoiding lighting. Finally, the fluorescent microscope from Nikon Eclipse 90i (Japan) was used to visualize and capture images.

### 2.7. ROS Assay

ROS assay kit was obtained from Beyotime Biotechnology (Shanghai, China). According to the instructions, cells were transfected for 48 hours and then treated with various concentrations of H_2_O_2_ or PBS for 2 hours. After washing with PBS, the cells were suspended in 500 *μ*l serum-free medium with 2,7-DCFH-DA (10 mM) for 60 min avoiding lighting, after which the cells were cleaned again with the same medium. The cells were excited at 488 nm, and the image was visualized at 525 nm using fluorescence microscopy from Olympus (Japan).

### 2.8. Protein Preparation

For protein preparation, the cells were lysed with cell lysates buffer (50 mM Tris, 137 mM NaCl, 1 mM EDTA, 10 mM NaF, 0.1 mM Na3VO4, 1% NP-40, 1 mM DTT, 10% glycerol, pH 7.8, with protease inhibitors provided by Roche (Switzerland)) and then centrifuged at 12000 rpm/min for 15 minutes at low temperature. And then protein quantification was used by BCA to ensure 40 *μ*g total protein in each sample.

### 2.9. Coimmunoprecipitation

For immunoprecipitation, after washing twice by PBS, H9c2 cardiomyocytes were lysed with flag lysis buffer. The lysates were mixed with relevant antibody and 30 *μ*l of magnetic beads for a night. Next, the mixture was washed with lysate and dissolved to 20 *μ*l 2×SDS-PAGE.

### 2.10. Western Blot Analysis

Firstly, protein samples were separated on 10% SDS polyacrylamide gel electrophoresis. And then, PVDF membranes provide by Millipore (USA) were used to transfer. After transferring, the membranes were blocked in TBS/T buffer with 5% BSA at room temperature. Finally, the membranes were put in to TBS/T buffer with 1% BSA, which diluted primary antibodies at 1 : 1000 in 4°C overnight. Next, the membranes were incubated with the diluted second antibody at 1 : 5000 for 2 hours homeothermy. After washing with TBS/T buffer for 45 minutes, the membranes were visualized and captured using enhanced chemiluminescence. Relative band intensity to control was measured by ImageJ software 1.46 (USA).

### 2.11. Statistical Analysis

All data reported was in the form of mean ± standard deviation and analyzed with Student's *t*-test or one-way analysis of variance (ANOVA), which was performed using SPSS 22.0 statistical software (USA). Values were considered significant as *p* < 0.05.

## 3. Results

### 3.1. H_2_O_2_ Increased Cul4a and Apoptosis-Related Protein and Dramatically Decreased the Viability of H9c2 Cardiomyocytes

**Figure 1:**
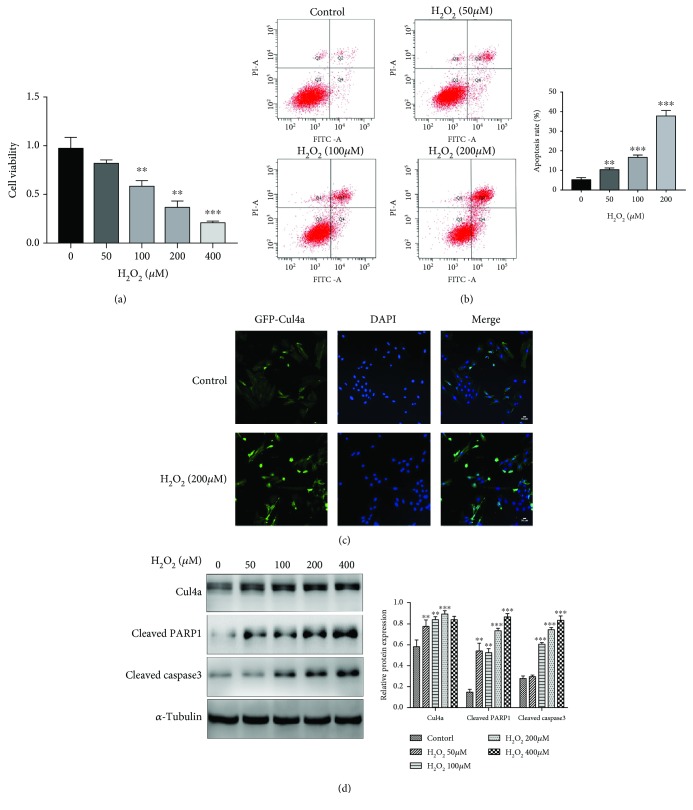
H_2_O_2_ increased Cul4a and apoptosis-related protein and decreased the viability of H9c2 cardiomyocytes. (a) H9c2 cells were untreated or treated with 50 *μ*M, 100 *μ*M, 200 *μ*M, and 400 *μ*M H_2_O_2_ for 2 h. Cell viability was assessed by CCK-8 assay. (b) Flow cytometry Annexin-FITC/PI showed that the apoptosis rate of cell increased gradually induced by gradient concentration of H_2_O_2_. (c) Immunofluorescence staining of H9c2 with Cul4a antibody (green) and DAPI for nuclei (blue) after treatment with 200 *μ*M of H_2_O_2_ for 2 h. (d) Western blot analysis of Cul4a, cleaved PARP1 and cleaved caspase3 expression after incubation with H_2_O_2_ (50 *μ*M, 100 *μ*M, 200 *μ*M, and 400 *μ*M) for 2 h; *α*-tubulin was used as a loading control. ^∗∗∗^*p* < 0.001, ^∗∗^*p* < 0.01, ^∗^*p* < 0.05; scale bar, 50 *μ*m. Data was expressed as means ± SD.

Given that few studies had demonstrated how Cul4a changed in oxidative stress-induced myocardial apoptosis, we took the initiative to determine the trend of the Cul4a expression in cardiomyocytes treated with H_2_O_2_ at a concentration of 0, 50, 100, 200, and 400 *μ*M for 2 hours. As shown in Figure 1, the expression of Cul4a, apoptotic marker proteins cleaved PARP1 and cleaved caspase3, increased obviously with concentration. Moreover, the cell viability notably decreased, and the apoptosis rate increased. Two hours after the treatment of 200 *μ*M H_2_O_2_, immunofluorescence imaging analysis was used to determine the Cul4a expression of the level and localization. Therefore, our results revealed that Cul4a may be crucial in oxidative stress-induced myocardial injury.

### 3.2. Cul4a Overexpression Inhibited H_2_O_2_-Induced Cytotoxicity, Apoptosis, and Apoptotic Marker Protein Expression

**Figure 2:**
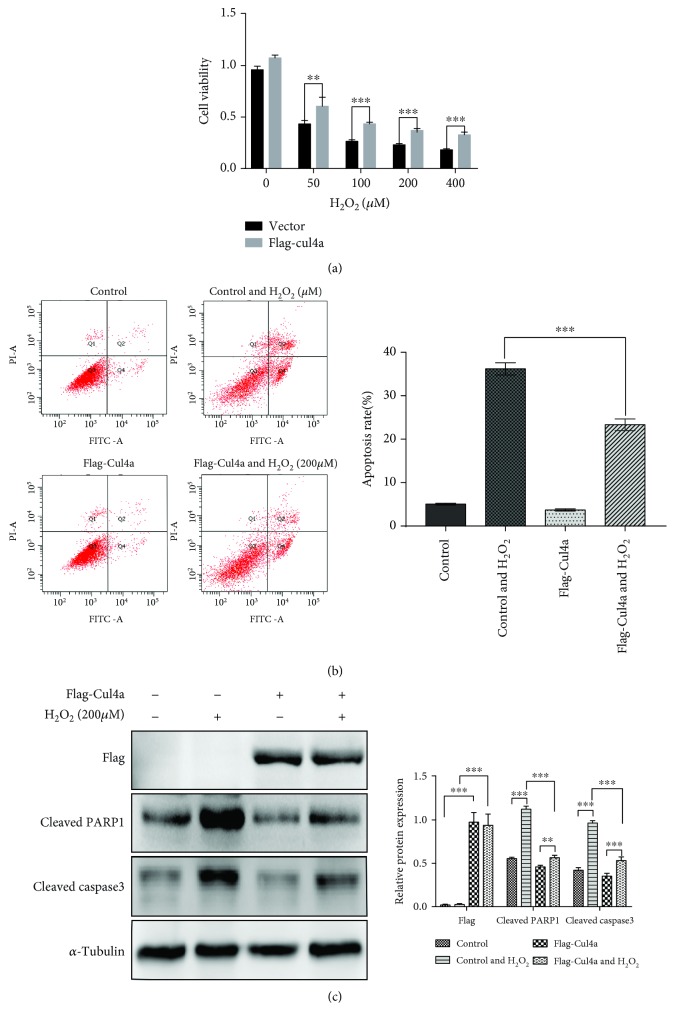
Cul4a overexpression inhibited H_2_O_2_-induced cardiomyocyte apoptosis. (a) H9c2 cells were untreated or treated with 50 *μ*M, 100 *μ*M, 200 *μ*M, and 400 *μ*M H_2_O_2_ for 2 h. CCK-8 assay results showed that overexpressed Cul4a decreased H_2_O_2_-induced cell cytotoxicity. (b) Flow cytometry Annexin-FITC/PI showed that overexpressed Cul4a reduced H_2_O_2_-induced cell apoptosis. (c) Overexpressed Cul4a significantly decreased H_2_O_2_-induced apoptotic marker proteins cleaved PARP1 and cleaved caspase-3 expression by western blot assay; *α*-tubulin was used as a loading control. ^∗∗∗^*p* < 0.001, ^∗∗^*p* < 0.01, ^∗^*p* < 0.05. Data was expressed as means ± SD. Cul4a: cullin 4a; PARP1: poly (ADP-ribose) polymerase-1.

To validate the effect of Cul4a in H_2_O_2_-induced myocardial injury, H9c2 cardiomyocytes were handled with Flag-Cul4a plasmid or empty plasmid for 48 hours, and then oxidative stress was induced with 200 *μ*M H_2_O_2_ for 2 hours. As shown in Figure 2, Cul4a overexpression inhibited H_2_O_2_-induced cytotoxicity, apoptosis, and apoptotic marker protein expression. Thus, the outcome indicated that Cul4a overexpression significantly inhibited oxidative stress-induced myocardial apoptosis.

### 3.3. Cul4a Knockdown Raised H_2_O_2_-Induced Cytotoxicity, Apoptosis, and Apoptotic Marker Protein Expression

**Figure 3:**
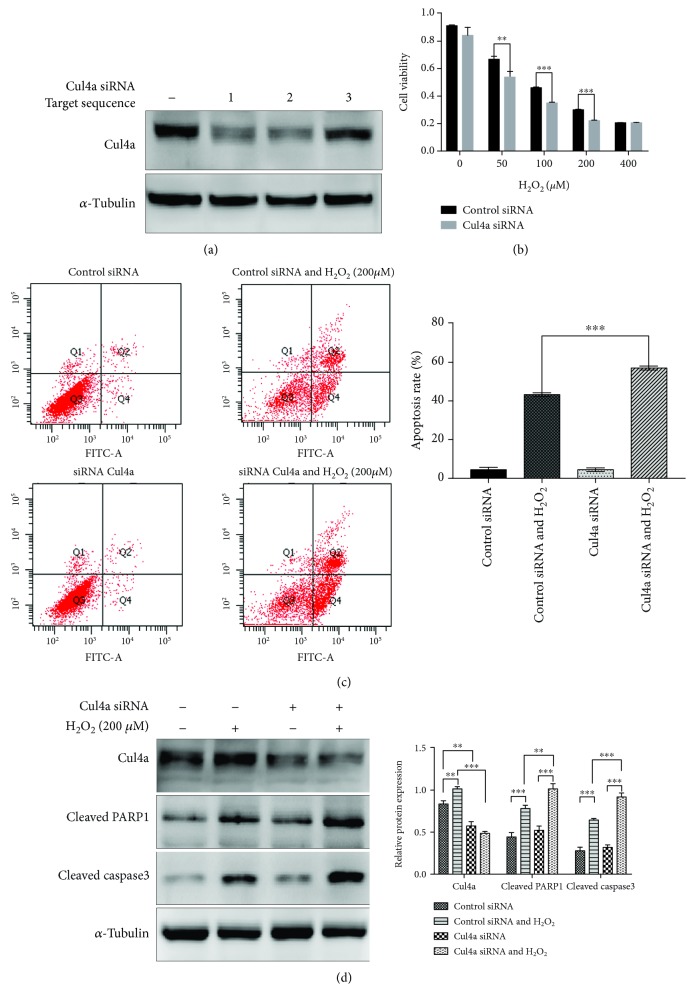
Cul4a knockdown aggravated H_2_O_2_-induced H9c2 cell apoptosis. (a) Efficient Cul4a knockdown was established successfully when compared to control siRNA-transfected cells, and Cul4a siRNA target sequence 2 has the highest knockdown efficiency. (b) H9c2 cells were untreated or treated with 50 *μ*M, 100 *μ*M, 200 *μ*M, and 400 *μ*M H_2_O_2_ for 2 h. CCK-8 assay results showed that Cul4a knockdown increased H_2_O_2_-induced cell cytotoxicity. (c) Flow cytometry Annexin-FITC/PI showed that Cul4a knockdown aggravated H_2_O_2_-induced cell apoptosis. (d) Knockdown Cul4a significantly enhanced H_2_O_2_-induced apoptotic marker proteins cleaved PARP1 and cleaved caspase-3 expression by western blot assay; *α*-tubulin was used as a loading control. ^∗∗∗^*p* < 0.001, ^∗∗^*p* < 0.01, ^∗^*p* < 0.05. Data was expressed as means ± SD. Cul4a: cullin 4a; PARP1: poly (ADP-ribose) polymerase-1.

In order to further explore the effects of Cul4a knockdown on H_2_O_2_-induced injury, we practiced siRNA knockdown of Cul4a. Cul4a siRNA target sequence 1, 2, and 3 were synthesized in order to choose the optimal efficiency.  Figure 3(a) showed, comparing to control siRNA-transfected cells, Cul4a knockdown was established successfully, and we chose Cul4a siRNA target sequence 2 for subsequent experiments owing to the highest knockdown efficiency. Then, we used control siRNA and Cul4a sequence 2 to transfect H9c2 cells for 48 hours, and subsequently, oxidative stress was induced with 200 *μ*M H_2_O_2_ for 2 hours.  Figure 3 demonstrated that Cul4a knockdown raised H_2_O_2_-induced cell cytotoxicity, apoptosis, and apoptotic marker proteins cleaved PARP1 and cleaved caspase3 expression. Therefore, these results proved Cul4a suppression promoted oxidative stress-induced myocardial apoptosis.

### 3.4. Cul4a Influenced H_2_O_2_-Induced ROS Formation

**Figure 4:**
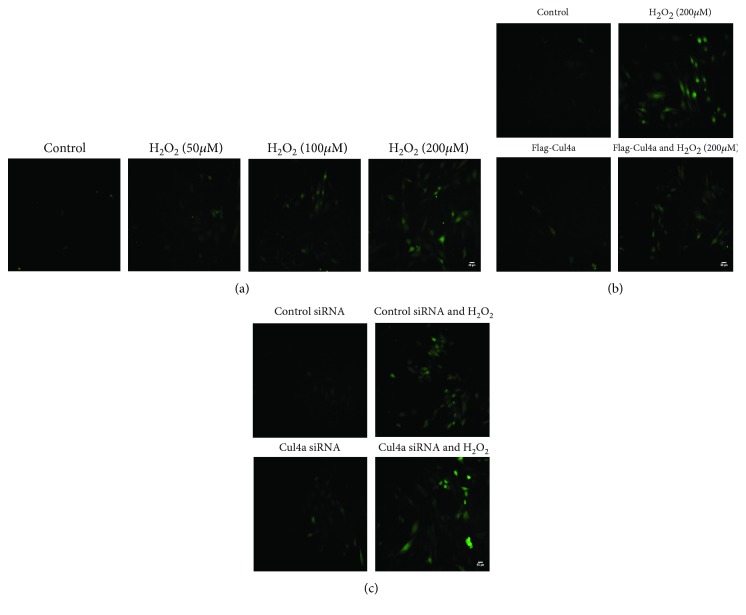
Cul4a influenced H_2_O_2_-induced ROS formation. (a) H9c2 cells were untreated or treated with 50 *μ*M, 100 *μ*M, and 200 *μ*M H_2_O_2_ for 2 h; ROS production increased gradually by using fluorescence microscopy. (b) Cul4a overexpression inhibited H_2_O_2_-induced ROS production by using fluorescence microscopy. (c) Cul4a knockdown enhanced H_2_O_2_-induced ROS production by using fluorescence microscopy. Scale bar = 50 *μ*m. Cul4a: cullin 4a.

To demonstrate the effects of Cul4a on oxidative stress, we exercised a ROS assay. Firstly, we verified that H_2_O_2_ can induce ROS production. We treated cardiomyocytes with H_2_O_2_ at a concentration of 0, 50, 100, and 200 *μ*M for 2 hours; the results in Figure 4(a) showed that the expression of ROS formation increased. Next, we established Cul4a overexpression and knockdown and treated the cells with 200 *μ*M H_2_O_2_ for 2 h; it was found that Cul4a overexpression hindered ROS generation as shown in Figure 4(b) and Cul4a suppression promoted ROS production as shown in Figure 4(c). This suggested that Cul4a could affect H_2_O_2_-induced ROS production, thereby mitigating cell damage caused by oxidative stress.

### 3.5. Identification of PARP1 as a New Cul4a-Binding Protein and the Interaction Was Enhanced under Oxidative Stress

**Figure 5:**
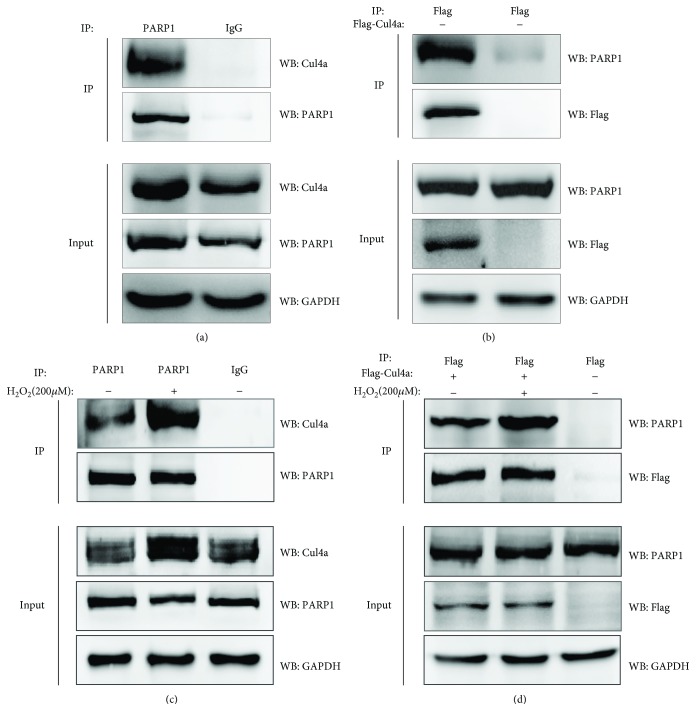
PARP1 was a novel Cul4a-interacting protein, and the interaction was enhanced under oxidative stress. (a) Coimmunoprecipitation (co-IP) and western blotting (IP-western) using anti-PARP1 antibody or negative control IgG and Protein A/G immunoprecipitation magnetic beads followed by anti-Cul4a western blot were performed to identify endogenous interaction between PARP1 and Cul4a. (b) Anti-Flag antibody and Protein A/G immunoprecipitation magnetic beads followed by anti-PARP1 western blot were performed to identify semiexogenous interaction between Flag-Cul4a and PARP1. (c) Endogenous interaction between PARP1 and Cul4a was enhanced by treatment of 200 *μ*M H_2_O_2_ for 2 h, which were evaluated by coimmunoprecipitation using anti-PARP1 antibody. (d) Semiexogenous interaction between Flag-Cul4a and PARP1 was enhanced by treatment of 200 *μ*M H_2_O_2_ for 2 h, which were evaluated by coimmunoprecipitation using anti-Flag antibody. GAPDH was used as a loading control. Cul4a: cullin 4a; PARP1: poly (ADP-ribose) polymerase-1; GAPDH: glyceraldehyde-3-phosphate dehydrogenase.

The above results indicated Cul4a operates positive effect in oxidative stress-induced myocardial cell injury. But the mechanism remained not clear. We identified PARP1 was a new Cul4a-interacting protein through applying coimmunoprecipitation. As shown in Figure 5, endogenous and semiexogenous Cul4a interacted with PARP1, and the interaction was enhanced after using by H_2_O_2_ treatment. Thus, it revealed apoptosis-related protein PARP1 can serve as a new binding protein of Cul4a, and the interaction was strengthened under oxidative stress. These results implied Cul4a may reduce damage by interacting with overactivated PARP1 in oxidative stress-induced myocardial cell injury.

### 3.6. Confirmed That Cul4a Could Decrease the Expression of PARP1

**Figure 6:**
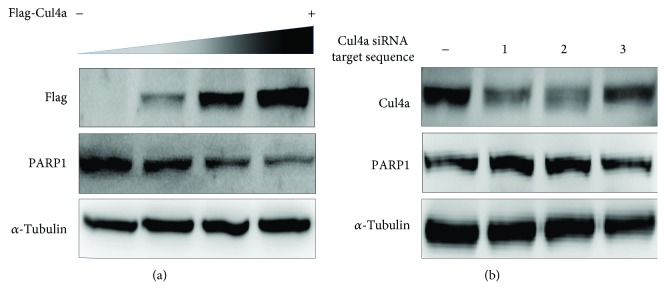
Cul4a can decrease the expression of PARP1. (a) Cul4a gradient overexpress with different amounts of plasmid in H9c2 cells, the expression of PARP1 decreased. (b) Knockdown Cul4a with control siRNA and Cul4a siRNA target sequence 1, 2, and 3, the expression of PARP1 with Cul4a siRNA target sequence 1 and 2 increased. *α*-Tubulin was used as a loading control. Cul4a: cullin 4a; PARP1: poly (ADP-ribose) polymerase-1.

Proceeding to sufficiently examine the effect of Cul4a and PARP1 binding on PARP1, we established Cul4a gradient overexpression with different amounts of plasmid in H9c2 cells. The expression of PARP1 was detected by western blot. As shown in Figure 6, with the increase in the expression of Cul4a, the PARP1 expression decreased, while the knockdown Cul4a showed the opposite effect, which implied that Cul4a can reduce PARP1.

## 4. Discussion

We discovered Cul4a as a novel protein that was involved in oxidative stress-induced myocardial apoptosis. Moreover, overexpression of Cul4a could obviously attenuate oxidative stress-induced cardiomyocyte apoptosis, and the knockdown of Cul4a showed just the opposite effect. We also found that Cul4a was a novel binding protein of PARP1 and reduced the expression of PARP1.

PARP1 is activated in order to repair DNA damage during cell damage; however, hyperactive PARP1 plays a negative role in cell apoptosis and even death after damage [12]. A study from Fouquerel et al. showed that PARP1 hyperactivation results in reduced glycolysis and ATP loss [13]. PARP1 activity has also been suggested to reduce respiration by direct influence on the enzymatic activity of mitochondrial proteins [14]. In oxidative stress, activated PARP1 can increase ROS production, which triggered the cell death [15] and can consume large amounts of ATP, ultimately leading to apoptosis [16]. PARP1 is widely involved in oxidative stress-related cardiovascular diseases [6, 7]. Recent studies have shown that hyperactive PARP1 exacerbates ischemic reperfusion injury in vivo and in vitro experiment. PARP1 activation undermines cell survival by preventing mitochondrial recovery after mPTP opening early in reperfusion [17], and PARP1 inhibitors can significantly alleviate oxidative stress-induced reperfusion injury [8, 18]. Hence, PARP1 plays an important role in regulating cardiovascular diseases. We identified PARP1 as a novel binding protein of Cul4a ubiquitin ligase in physiological state, and the interaction is significantly increased in oxidative stress state. It implies that Cul4a is a novel protective factor in oxidative stress-induced myocardial injury by interacting with PARP1 and degrading it.

For the first time, we found that Cul4a was elevated in oxidative stress-induced myocardial apoptosis. Further, Cul4a overexpression could reduce the damage, whereas knockdown could aggravate it. At the same time, Cul4a also influenced the amount of ROS. Cul4a is an E3 ubiquitin ligase of significance in DNA replication, cell cycle regulation, and genomic instability [11]. Cul4a is abnormally expressed in various cancers, and many studies have shown that it is closely related to the proliferation and invasion of lung cancer [19], gastric cancer [20], and prostate cancer [21]. Cul4a is vital for cell survival. Waning et al. reported that the deletion of Cul4A can increase the expression of Cul4A ligase target Cdt 1 and P27, which results in apoptosis [22]. In addition, Cul4A can be central in the regulation of the cellular levels of PNKP and hence in the repair of oxidative DNA damage [23]. There has been a report that zebrafish Cul4a modulates cardiac and forelimb development by upregulating the tbx5a expression [24]. However, the role of Cul4a in cardiovascular disease has been little reported. Cul4a acts as the core of the CRLs E3 ubiquitin ligase, and its complex can bind multiple substrates [9]. Here, we reported that a new binding protein PARP1 could be combined with Cul4a, and in the case of oxidative stress, this binding was significantly enhanced. We also found the regulation of Cul4a controlled the expression of PARP1. Based on the information above, we hypothesized that Cul4a can be elevated and inhibit apoptosis by binding PARP1 to degrade excess PARP1. These findings may provide a target for the treatment of reperfusion injury.

Our study shows that Cul4a is involved in oxidative stress-induced myocardial apoptosis and PARP1 is a new binding protein to Cul4a ubiquitin ligase, which may become a new therapeutic target in reperfusion injury. However, we acknowledge that there are some limitations in our study. Cul4a knockdown or Cul4a overexpression mice have not been performed to analyze the effect of Cul4a on reperfusion injury. In addition, although our study is the first to report that Cul4a is involved in oxidative stress-induced myocardial injury, whether Cul4a is associated with other cardiovascular diseases is still unclear. Moreover, as the fact has been confirmed that Cul4a degrades target proteins through ubiquitin ligase, it is attractive to explore the detailed mechanism about how Cul4a degraded PARP1 by ubiquitination. Therefore, further experiments are needed.

## 5. Conclusions

We have demonstrated that Cul4a is involved in oxidative stress-induced myocardial cell apoptosis and PARP1 is a novel interaction protein for Cul4a. When Cul4a is overexpressed, PARP1 decreases, so does the generation of ROS, which can obviously attenuate oxidative stress-induced cardiomyocyte injury.

## Data Availability

The data used to support the findings of this study are included within the article.
